# Prediction of the Effect of Sleep Deprivation on Response Inhibition via Machine Learning on Structural Magnetic Resonance Imaging Data

**DOI:** 10.3389/fnhum.2018.00276

**Published:** 2018-07-10

**Authors:** Rui Zhao, Xinxin Zhang, Yuanqiang Zhu, Ningbo Fei, Jinbo Sun, Peng Liu, Xuejuan Yang, Wei Qin

**Affiliations:** ^1^Engineering Research Center of Molecular and Neuro Imaging of the Ministry of Education, School of Life Science and Technology, Xidian University, Xi’an, China; ^2^Department of Radiology, Xijing Hospital, The Fourth Military Medical University, Xi’an, China

**Keywords:** inhibitory control, stop-signal task, prediction, VBM, linear regression

## Abstract

Sleep deprivation (SD) impairs the ability of response inhibition. However, few studies have explored the quantitative prediction of performance impairment using Magnetic Resonance Imaging (MRI) data. In this study, structural MRI data were used to predict the change in response inhibition performance (ΔSSRT) measured by a stop-signal task (SST) after 24 h of SD in 52 normal young subjects. For each subject, T1-weighted MRI data were acquired and the gray matter (GM) volumes were calculated using voxel-based morphometry (VBM) analysis. First, the regions in which GM volumes correlated with ΔSSRT were explored. Then, features were extracted from these regions and the prediction process was performed using a linear regression model with four-fold cross-validation. We found that the GM volumes of the left middle frontal gyrus (L_MFG), pars opercularis of right inferior frontal gyrus (R_IFG), pars triangularis of left inferior frontal gyrus, pars opercularis of right rolandic area, left supplementary motor area (L_SMA), left hippocampus, right lingual gyrus, right postcentral gyrus and left middle temporal gyrus (L_MTG) could predict the ΔSSRT with a low mean square error of 0.0039 ± 0.0011 and a high Pearson’s correlation coefficient between the predicted and actual values of 0.948 ± 0.0503. In conclusion, our results demonstrated that a linear combination of structural MRI data could accurately predict the change in response inhibition performance after SD. Further studies with larger sample sizes and more comprehensive sample may be necessary to validate these findings.

## Introduction

Inhibitory control, also known as response inhibition, is the process of being able to rapidly cancel planned or ongoing behaviors. It is indispensable for self-regulation, and is an important prerequisite for higher-order executive functions (Diamond, 2013). Poor response inhibition may profoundly interfere with the requirements of everyday life. Inhibitory control is also an important function in individual daily activities, such as being able to stop driving upon seeing a pedestrian unexpectedly cross the road (Lee and Hsieh, 2017). However, sleep deprivation (SD) for one night can impair the performance of inhibitory control (Chuah et al., 2006; Zhao et al., 2018). Therefore, a decrease in response inhibition ability after SD could have deleterious outcomes, and being able to predict the effect of SD on inhibitory control could help avoid danger in the future.

Predictive data mining has become a much-discussed area of research over the last decade. Recent neuroimaging studies have started to use machine-learning techniques to predict cognitive or behavioral performance from functional magnetic resonance imaging (fMRI) data (Eichele et al., 2008; Aharoni et al., 2013; Finn et al., 2015) and diffusion tensor imaging data (Ellingson et al., 2015; Park et al., 2015). Furthermore, Mwangi et al. (2011) used structural T1-weighted MRI data to predict the severity of major depressive disorder illness. Supekar et al. (2013) used morphometry and functional connectivity to predict math-tutoring outcomes in primary-grade school children. Redlich et al. (2016) used gray matter (GM) volumes to predict electroconvulsive therapy response. These studies suggest that certain clinical features and cognitive performance can be predicted from structural MRI data. Therefore, we hypothesized that the change in response inhibition performance after SD could be predicted from regional GM volumes. However, we are not aware of any other published studies that have evaluated the prediction of GM volumes to the response inhibition performance impairment after SD.

Previous fMRI (Li et al., 2006; Chevrier et al., 2007; Cohen et al., 2010; Congdon et al., 2010; Kenner et al., 2010; Erika-Florence et al., 2014), lesion (Aron et al., 2003; Floden and Stuss, 2006; Picton et al., 2007) and transcranial magnetic stimulation (TMS) studies (Chambers et al., 2006, 2007; Chen et al., 2009; Zandbelt et al., 2013) have revealed that the right inferior frontal gyrus (IFG) is a critical region for the successful inhibitory control (Aron et al., 2004, 2014). Recent evidence suggests that the IFG and supplementary motor area (SMA) might coordinate inhibition via direct white-matter tract (Aron et al., 2007). Furthermore, the SMA are implicated from fMRI studies (Aron and Poldrack, 2006; Xue et al., 2008; Chambers et al., 2009; Cai et al., 2014) and brain stimulation studies (transcranial direct current stimulation and TMS; Yu et al., 2015; Lee et al., 2016). Additional brain regions, such as middle frontal gyrus (MFG), occipital cortex and middle temporal gyrus (MTG) have also been reported to be involved in inhibitory control (Aron and Poldrack, 2006; Congdon et al., 2010, 2014; Galván et al., 2011; Swick et al., 2011). These studies suggest that these regions may be the “stopping network” related with inhibitory control. Therefore, we hypothesized that the regions in stopping network would predict the change in response inhibition performance after SD.

In the present study, we attempted to use voxel-based morphometry (VBM) analysis to investigate whether regional GM volumes could predict ΔSSRT (the change in the rate of response inhibition measured by a stop-signal task (SST) before and after SD). First, we explored the relationship between GM volume and ΔSSRT using voxel-wise analysis. Then, we extracted the GM volumes of regions that were negative correlated with ΔSSRT. Finally, we used a linear regression model to investigate the performance of these GM volumes in the prediction of ΔSSRT.

## Materials and Methods

### Subjects

Fifty-seven right-handed healthy subjects were recruited from Xidian University in this study. They were undergraduate and master students of Xidian University. All subjects had no history of self-reported medical, psychiatric, neurologic, or sleep disorders, and were free of any abused alcohol or drugs. Subjects who presented an extreme morning or extreme evening type as assessed by a questionnaire (Horne and Ostberg, 1975), were excluded. They habitually maintained normal sleep schedules of 7–9 h per night, between 10:00 pm and 8:00 am.

Two subjects opted out of this study after SD. Another three subjects were excluded because of the abnormality in brain structure. Therefore, the final analyzed group consisted of 52 subjects (mean age 19.53 ± 1.62 years, range 17–23; 26 males and 26 females). All participants declared that they did not smoke or consume any stimulants, medications, alcohol or caffeine for at least 24 h prior to the formal experiment. All research procedures were conducted in accordance with the Declaration of Helsinki and approved by the institutional research ethics committee of the Xijing Hospital of the Fourth Military Medical University. All subjects provided written informed consent prior to participation and were compensated for their time.

### Experimental Procedure

**Figure 1:**
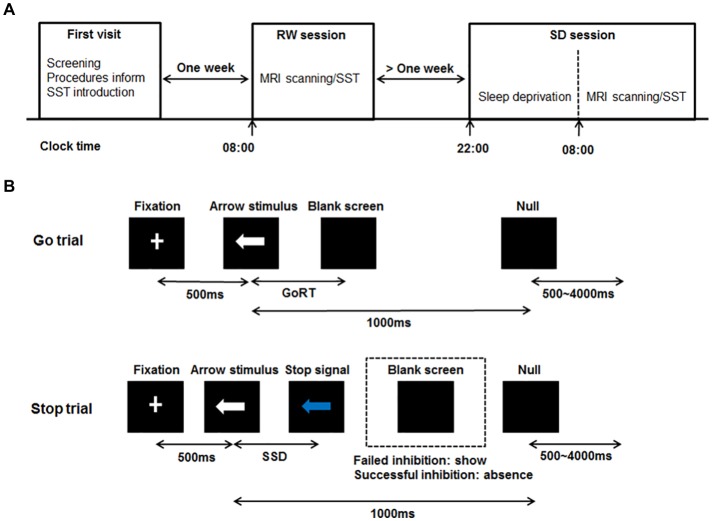
Schematic diagram of the experimental procedure and the stop-signal task (SST). **(A)** Schematic showing the order of the experimental procedure. For the first visit, subjects underwent the screening process, were informed of the experimental procedures and were given instructions about the SST. After 1 week, subjects administered the simultaneous magnetic resonance imaging (MRI) scanning/SST twice at 8:00 am in the second visit and third visit respectively, once after RW and once after 24 h of SD. The RW and SD sessions were administered in a randomized, cross-over fashion with at least 1 week apart. For the SD session, subjects were monitored in the laboratory from 10:00 pm to 8:00 am to prevent them from falling asleep. **(B)** Schematic of the SST including Go trial and Stop trial. All trials started with 500 ms white cross fixation in the center of the black background screen. Then, a left- or right-pointing arrow stimulus was displayed on a computer screen. On Go trial, subjects were instructed to press the button as soon as possible within 1000 ms. If subjects responded, the arrow stimulus disappeared, and the blank screen was shown. The interval between the arrow stimulus and blank screen was the GoRT. On Stop trail, subjects were instructed to stop pressing the button when a Stop signal (the white arrow was changed to blue) was presented after SSD. If subjects inhibited their response (Successful inhibition), the Stop signal remained onscreen for the duration of 1000 ms—SSD. If subjects pressed the button (Failed inhibition), the Stop signal disappeared, and the blank screen was shown. For both trials, the null events, consisting of blank screen, were also shown between every trial with the duration ranging between 500 ms and 4000 ms. RW, rested wakefulness; SD, sleep deprivation; GoRT, reaction time on correct Go trials; SSD, stop-signal delay.

All subjects were scheduled for three visits to the laboratory. The schematic of this experimental procedure can be seen in Figure 1A. For the first visit, subjects visited the laboratory, underwent the screening process, were informed of the experimental procedures and were given instructions about the SST. After 1 week, subjects who met the inclusion criteria administered the SST twice at 8:00 am in the second visit and third visit respectively, once after rested wakefulness (RW) and once after 24 h of SD. These two states were administered in a randomized, cross-over fashion with at least 1 week apart to minimize possible residual effects of SD on cognition (Van Dongen et al., 2003). Some subjects showed poor sleep when they slept normally in the laboratory during the pre-experiment. Furthermore, during the RW night, no action was performed for subjects, and we didn’t perform any operation on subjects. Therefore, we arranged with subjects to sleep normally at home during the RW night. In order to ensure that subjects slept well during the RW night at home, they finished the sleep diary after waking up. If one’s sleep quality was poor, he/she would not perform the SST on this day. We would arrange the experiment next time again. Subjects with well sleep reported to the laboratory at 7:30 am and performed the SST while being scanned with fMRI at 8:00 am. During the SD night, subjects were monitored in the laboratory from 10:00 pm to 8:00 am to prevent them from falling asleep. They were allowed to engage in non-strenuous activities such as reading and watching videos. Each subject completed the SST in the MRI scanner at 8:00 am. Directly before each scanning session, each subject performed a training session of the SST. After performed the SST during the second visit, the structure MRI data of each subject was acquired.

### The Stop-Signal Task

**Figure 2:**
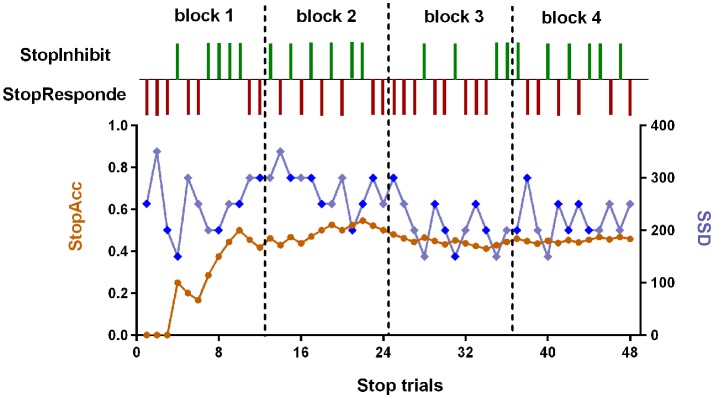
The dynamic changes of SSD and StopAcc across Stop trials of one subject. The green vertical lines represent the successful inhibition (StopInhibit). The purplish red vertical lines represent the failed inhibition (StopResponde). The brown curve (left ordinate) represents the accuracy of Stop trials (StopAcc). The baby blue (right ordinate) curve represents the SSD value. To reduce participants’ anticipation of stimuli, two staircases were used and respectively started with SSD values of 250 and 350 ms (the first two values in the baby blue curve). The dark blue diamond points represent the first staircase with start value of 250 ms. The remaining baby blue diamond points represent the second staircase with start value of 350 ms. If the subject inhibited successfully on a Stop trial, the inhibition was made more difficult on a subsequent Stop trial by increasing the SSD by 50 ms; if the subject did not successfully inhibit, the inhibition was made easier by decreasing the SSD by 50 ms. There were four blocks with 48 Stop trials in total. In each block, six Stop trials corresponded to the first staircase, while the other six Stop trials corresponded to the second staircase. The order of the two staircases was randomized trial-by-trial. For continuity, the start values of the two staircases in block 2 were the last values in block 1, and so on. We can see from this figure that the StopAcc tended to be stable and was about 50% via the dynamical changing the SSD.

The SST was adapted from previous published works (Logan, 1994; Aron and Poldrack, 2006). The schematic of this task can be seen in Figure 1B. There were two trial types: Go (75% of trials) and Stop (25% of trials). All trials started with 500 ms white cross fixation in the center of the black background screen. Then, a left- or right-pointing arrow was displayed on a computer screen. On Go trials, subjects were instructed to press the right button as soon as possible with their right middle finger within 1000 ms if a right-pointing arrow was presented, and to press the left button with their right index finger if a left-pointing arrow was displayed. If subjects responded, the arrow stimulus disappeared from the center of the computer screen, followed by the blank screen. The null events, consisting of blank screen, were also shown between every trial. The duration of null events ranged between 500 ms and 4000 ms, with a mean of 1000 ms. On Stop trails, subjects were instructed to stop pressing the button when a Stop signal (the white arrow was changed to blue) was presented after a particular delay (stop-signal delay, SSD) subsequent to the arrow stimulus. If subjects inhibited their response, the Stop signal remained onscreen for the duration of 1000 ms—SSD. If subjects pressed the button, the Stop signal disappeared from the center of the computer screen, followed by the blank screen. Similarly, the null events were also imposed between every trial, with the duration ranged between 500 ms and 4000 ms. The SSD changed dynamically throughout the experiment: if the subject inhibited successfully on a Stop trial, the inhibition was made more difficult on a subsequent Stop trial by increasing the SSD by 50 ms; if the subject did not successfully inhibit, the inhibition was made easier by decreasing the SSD by 50 ms. This procedure (also named a staircase procedure) was performed to achieve approximately 50% accuracy of Stop trials (StopAcc) and control the difficulty level across subjects. To reduce participants’ anticipation of stimuli, two staircases were used and respectively started with SSD values of 250 and 350 ms. There were two runs in per state. Each 4.5 min run included two blocks, giving a total of 36 Go trials and 12 Stop trials in each block. Each Stop trial corresponded to one staircase. Therefore, six Stop trials corresponded to the staircase commencing with an SSD of 250 ms, while the other six Stop trials corresponded to the staircase commencing with 350 ms. Each staircase moved six times within each block. The order of the two staircases was randomized trial-by-trial. The dynamic changes of SSD and StopAcc across 48 Stop trials are described in Figure 2.

### Behavioral Analysis

The stop signal reaction time (SSRT) was the primary measure of interest in the stop-signal task. It reflects the individual stop latency, and has been used as an index of an individual’s inhibitory control, with a shorter SSRT indicating better inhibitory control. SSRT was calculated using a quantile method, which corrected for deviations from 50% successful inhibition (Logan, 1994; Band et al., 2003). First, all reaction times (GoRT) on correct Go trials were arranged in ascending order. The RT corresponding to the proportion of failed inhibitions (1 − StopAcc) was then determined, termed the quantile RT. For example, if the StopAcc for a particular subject was 40%, the quantile RT would be the RT for which 40% of trials were faster than the quantile RT and 60% of trials were slower. SSRT was estimated as the difference between the quantile RT and the average SSD. Finally, the change in the rate of SSRT (ΔSSRT) was calculated according to the following:

$$\text{Δ}\text{SSRT = }\frac{{\text{SSRT}}_{\text{SD}}-{\text{SSRT}}_{\text{RW}}}{{\text{SSRT}}_{\text{RW}}}$$

### MRI Data Acquisition

MRI data were obtained on a 3T GE MR750 scanner at Department of Radiology, Xijing Hospital, The Fourth Military Medical University, Xi’an, China. A standard 8-channel head coil was used together with a restraining foam pad to minimize head motion and diminish scanner noise. To perform anatomical morphometric analyses, structural high-resolution 3D T1-weighted data were acquired in each subject using magnetization-prepared rapid-acquisition gradient echo sequence (MPRAGE; repetition time = 8.2 ms; echo time = 3.18 ms; field of view = 256 × 256 mm^2^; matrix = 512 × 512; in-plane resolution = 0.5 × 0.5 mm^2^; slice thickness = 1 mm; 196 sagittal slices; flip angle = 9°).

### Voxel-Based Morphometry Analysis

The VBM analysis was performed using the openware FSL version 5.0.4^1^ (Smith et al., 2004; Woolrich et al., 2009; Jenkinson et al., 2012). Data processing was divided into six major steps. (1) All individual T1-weighted images were brain-extracted using the brain extraction tool (BET; Smith, 2002) and visually inspected by an experienced neurologist to remove any leftover non-brain tissue. (2) Brain extracted images were segmented into different tissue types (GM, White Matter and cerebrospinal fluid) using the FMRIB’s Automated Segmentation Tool (FAST; Zhang et al., 2001). (3) The resulting GM images were registered to the GM ICBM-152 template using the affine registration tool FLIRT (Jenkinson and Smith, 2001; Jenkinson et al., 2002), followed by the nonlinear registration tool FNIRT^2^. The resulting images were averaged to create a symmetric, study-specific GM template. (4) The segmented individual GM images were then non-linearly registered to the study-specific GM template and modulated using the Jacobian of the warp field. (5) These modulated registered GM images were smoothed by a Gaussian kernel of 3 mm (e.g., sigma = 3 mm), with a full-width half-maximum (FWHM) of ~7 mm. (6) Finally, to explore the relationship between GM volume and ΔSSRT, voxel-wise whole-brain regression analysis was performed using a general linear model. Nonparametric statistics were performed using the “randomize” with 5000 permutations and family wise error (FWE) correction for multiple comparisons.

### Feature Extraction

We extracted features for the prediction analysis from the results of the VBM analysis. As the nature of a predictive analysis includes a built-in guard against false positives (Finn et al., 2015), we used an uncorrected threshold to determine the voxels which GM volumes were negative correlated with ΔSSRT. To explore the effect of different thresholds on the prediction, a range of uncorrected thresholds were chosen (*p* < 0.05, 0.01, 0.005 and 0.001, with a minimum of 10 voxels). The GM volumes of brain regions above the threshold were extracted and averaged according to the Automated Anatomical Labeling (AAL) template. The mean GM volumes of these brain regions were then calculated and regarded as candidate features.

### Feature Selection

In traditional linear regression analysis, the number of independent variable should be less than the sample size. However, in some cases, the dimensions of the above candidate features were more than the sample size, which suggest that there are correlations among some candidate features. Therefore, it was necessary to reduce the feature dimensions to impair the collinearity. Least Absolute Shrinkage and Selection Operator (LASSO), a commonly used method of dimensional reduction, was performed to make the variable selection (Tibshirani, 1996). LASSO estimates the regression coefficients through an ℓ1-norm penalized least-squares criterion and minimizes the residual sum of squares with an ℓ1 penalty on the regression coefficients (Waldmann et al., 2013). In this method, the regularization parameter λ controls the trade-off between data fitting and sparsity. The optimal value of λ was determined by a 10-fold cross-validation. The features with non-zero regression coefficients were then selected as the most promising candidate features. This procedure was conducted using the “glmnet” function in the “glmnet” package of the R project software^3^ (R Development Core Team, 2011).

### Prediction Analysis

**Figure 3:**
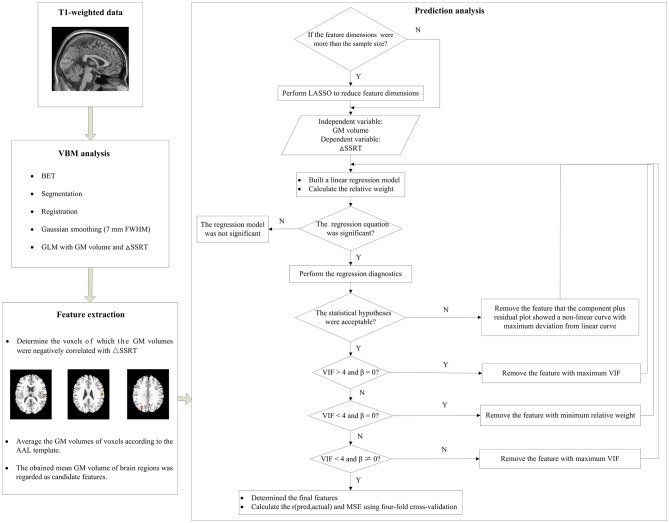
A schematic diagram of the prediction analysis pipeline. First, the T1-weighted data underwent the voxel-basedmorphometry (VBM) analysis to find the relationship between gray matter (GM) volume and ΔSSRT. Second, we extracted features from the results of the VBM analysis, and determined the voxels which GM volumes were negative correlated with the ΔSSRT at an uncorrected threshold. The GM volumes of voxels were then averaged according to the automated anatomical labeling (AAL) template. The obtained mean GM volumes of brain regions were regarded as candidate features. Third, we used these candidate features to predict the ΔSSRT with a linear regression model. If the feature dimensions were higher than the sample size, we performed least absolute shrinkage and selection operator (LASSO) to reduce the feature dimensions before building the linear regression model; otherwise, we directly built the linear regression model. We then evaluated the applicability of this model according to the following conditions: (1) the regression model was significant; (2) the linear regression model met the statistical hypotheses (normality, independence of errors, linearity, and homoscedasticity); (3) the regression coefficients (β) of all features were significant; (4) the features had no multicollinearity. If the model met all of the conditions, we calculated the r(pred, actual) and mean square error (MSE) using the final features; otherwise, features were removed one by one until the model met all the conditions.

A machine learning approach was used to examine the predictive ability of the GM volumes of brain regions. This approach involved cross-validation (Cohen et al., 2010; Supekar et al., 2013) combined with linear regression. A schematic diagram of the analysis pipeline is illustrated in Figure 3. The R project software was used to perform this analysis. First, the above most promising candidate features were input into a linear regression model as independent variables, with the ΔSSRT values as the dependent variable. A relative weight analysis was also performed to measure the relative importance of the independent variables (Johnson and Lebreton, 2004; Johnson, 2004; LeBreton and Tonidandel, 2008). The relative importance refers to the proportionate contribution of each independent variable to the coefficient of determination (*R*^2^), considering both the unique contribution of each independent variable by itself, and its incremental contribution when combined with the other independent variables in the regression equation. Then, we manually and gradually selected the most predictive features from the most promising candidate features via repeatedly examining the applicability of this linear regression model.

We evaluated the applicability of this model in three aspects. (1) We tested the significance of the regression equation with an F-test. If the regression model was not significant, the features would not explain the ΔpSSRT. (2) Regression diagnostics were performed using the “gvlma” function in the “gvlma” package to determine whether the linear regression model met the statistical hypotheses: normality, independence of errors, linearity and homoscedasticity. If the statistical hypotheses were not acceptable, we used the “qqPlot,” “durbinWatsonTest,” “crPlots,” and “ncvTest” functions to test which assumptions were not satisfied. In most cases, the normality, linearity and/or homoscedasticity assumptions were not satisfied. It would be possible to perform power transformations to the dependent variable and independent variables to adjust the model. However, power transformation was a cautious approach, and ΔSSRT had negative values, which would limit this method. Therefore, we removed the feature for which the component-plus-residual-plot showed a non-linear curve with maximum deviation from the linear curve, and built a new linear regression model without this feature. (3) We tested the significance of regression coefficients (β) with *t*-tests for each independent variable and evaluated the multicollinearity according to the variance inflation factor (VIF) using the “vif” function (Kabacoff, 2015). If the β of an independent variable was not significant, it meant that this independent variable did not have a remarkable effect on the dependent variable, and could be deleted from the regression model. If VIF was less than 4, there was no evidence of a multicollinearity problem (Kabacoff, 2015). In general, deleting some of the variables is a very important method for dealing with the multicollinearity problem. Therefore, we removed certain variables with VIF > 4. If the VIFs of some independent variables were above 4, the independent variable with the maximum VIF was removed. If VIF was under 4 but the β of some independent variables was not significant, the independent variable with the minimum relative weight was removed.

After the most predictive features were determined, the linear regression model was built. Four-fold cross-validation was performed to assess the prediction performance of this regression model, as it prevents the overfitting that can occur when leave-one-out cross-validation is used with small sample sizes (Cohen et al., 2010; Supekar et al., 2013). In brief, data were randomly divided into four groups. A linear regression model was built using three of the groups (i.e., training set). The left-out group, which served as the test set, was then predicted using this model, and the predicted value was obtained. This procedure was repeated four times with different test sets. Then, the Pearson’s correlation coefficient between the predicted values of the trained linear regression model in the test set and the actual values, termed r(pred, actual), was calculated. The average of the squares of the difference between the predicted values and the actual values, named MSE, was also computed. These two measurements were used as measures of how well the independent variables predicted the dependent variable, with r(pred, actual) = 1 and MSE = 0 being the most accurate prediction model. This procedure was repeated 1000 times to obtain a robust estimate of prediction performance. Finally, the mean r(pred, actual) and mean MSE were calculated, and the Pearson correlation between r(pred, actual) and MSE across the 1000 repetitions was computed. In order to explore the overfitting, we also calculated the predicted values of the trained linear regression model in the training set, and computed the mean MSE of training set. If the MSE of training set was much larger than that of test set, the model was highly likely overfitted. If the MSE of training set was much less than that of test set, the model was highly likely underfitted. If there was a little difference between the two MSEs, the model was appropriate fitted.

## Results

### Behavioral Results

**Figure 4:**
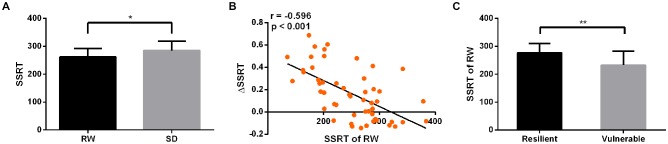
Behavioral results. After SD, subjects showed significantly poorer performance on SSRT (*p* = 0.03, **A**). The SSRT of RW was significantly negative correlated with ΔSSRT (*r* = −0.596, *p* < 0.001, **B**). The SSRT of RW in vulnerable group was significantly faster than that in resilient group (*p* = 0.004, **C**). *p < 0.05; **p < 0.01.

After SD, the StopAcc did not significantly differ from RW session (39.42 ± 5.23% after RW vs. 38.47 ± 8.35% after SD, *p* > 0.05, mean ± standard deviation, throughout). However, the subjects showed significantly poorer performance on the SSRT when they were sleep-deprived (261.41 ± 30.78 ms after RW vs. 285.14 ± 33.38 ms after SD, *p* = 0.03, Figure 4A). The ΔSSRT ranged from −0.144 to 0.687 (0.149 ± 0.241). Furthermore, we found that the RW SSRT was significantly negative correlated with ΔSSRT (*r* = −0.596, *p* < 0.001, Figure 4B). These results indicated that subjects with a faster SSRT after RW might show a greater change in SSRT after SD. According to this result, we hypothesized that subjects with better inhibition performance in the RW state would be more vulnerable to SD. To verify this hypothesis, we divided the 52 subjects into two groups (resilient and vulnerable groups) based on a median split of the average ΔSSRT. This dichotomization resulted in a total of 26 resilient subjects with lower ΔSSRT, and 26 vulnerable subjects with higher ΔSSRT. The age and gender of the two groups were matched (*p* = 0.614, two-sample *t*-test, for age; *p* = 0.328, chi-square test, for gender). We found that the mean RW SSRT of the vulnerable group was significantly faster than that of the resilient group (*p* = 0.004, 276.9 ± 7.89 ms in vulnerable group vs. 232.7 ± 12.06 ms in resilient group, Figure 4C). These findings confirmed the hypothesis that subjects with better inhibition performance in the RW state are more vulnerable to SD.

### Correlations Between ΔSSRT and GM Volumes

No significant correlation was observed between GM volume and ΔSSRT in any brain regions at a value of *p* < 0.05 corrected for multiple comparisons using FWE correction. For uncorrected thresholds of *p* < 0.05, *p* < 0.01, *p* < 0.005, and *p* < 0.001 with a minimum of 10 voxels, there were respectively 61, 24, 13 and 4 regions in which GM volumes were negative correlated with ΔSSRT (Supplementary Tables S1–S4).

### Prediction Results

**Figure 5:**
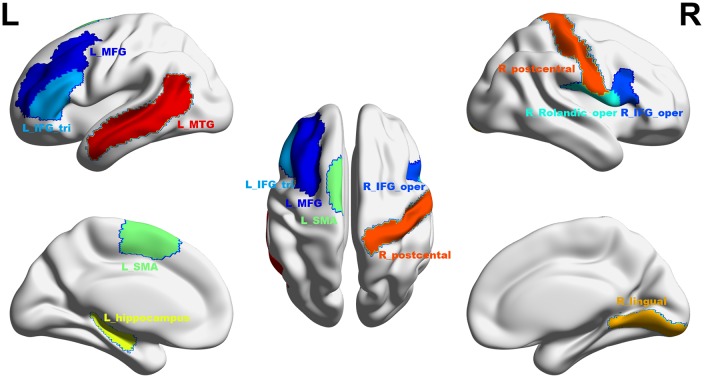
The final selected regions at threshold *p* < 0.05. Nine regions were selected: L_MFG, left middle frontal gyrus (shown in dark blue); R_IFG_oper, pars opercularis of right inferior frontal gyrus (blue); L_IFG_tri, pars triangularis of left inferior frontal gyrus (sky blue); R_Rolandic_oper, pars opercularis of right rolandic area (cyan); L_SMA, left supplementary motor area (mint green); L_hippocampus, left hippocampus (yellow); R_lingual, right lingual gyrus (saffron yellow); R_postcentral, right postcentral gyrus (orange red); L_MTG, left middle temporal gyrus (red). L, left; R, right. This figure was constructed using the BrainNet Viewer (http://www.nitrc.org/projects/bnv/; Xia et al., 2013).

**Figure 6:**
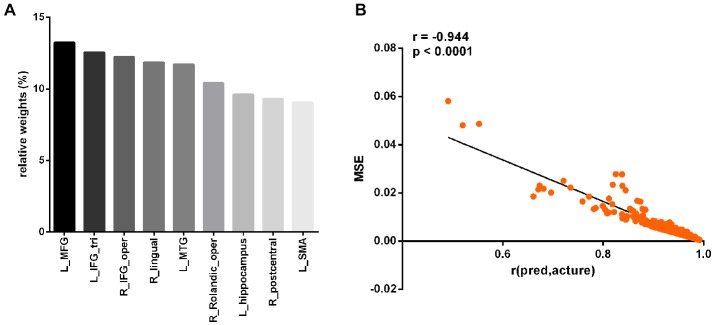
Prediction performance of the linear regression model with the nine regions. The relative weights of the nine regions **(A)**. The correlation between the r(pred, actual) and MSE **(B)**. L_MFG, left middle frontal gyrus; L_IFG_tri, pars triangularis of left inferior frontal gyrus; R_IFG_oper, pars opercularis of right inferior frontal gyrus; R_lingual, right lingual gyrus; L_MTG, left middle temporal gyrus; R_Rolandic_oper, pars opercularis of right rolandic area; L_hippocampus, left hippocampus; R_postcentral, right postcentral gyrus; L_SMA, left supplementary motor area.

At a significance threshold of *p* < 0.05, 61 brain regions were identified from the results of the VBM. LASSO was performed on these 61 regions and 14 were selected as the input for the linear regression model: the left precentral gyrus (L_precentral), left middle frontal gyrus (L_MFG), pars opercularis of right inferior frontal gyrus (R_IFG_oper), pars triangularis of left inferior frontal gyrus (L_IFG_tri), pars opercularis of right rolandic area (R_Rolandic_oper), left supplementary motor area (L_SMA), left hippocampus (L_hippocampus), left parahippocampal gyrus (L_parahippocampal), right lingual gyrus (R_lingual), left superior occipital gyrus (L_SOG), left inferior occipital gyrus (L_IOG), right postcentral gyrus (R_postcentral), right angular gyrus (R_angular) and left middle temporal gyrus (L_MTG). The GM volumes of these regions were entered as independent variables and ΔSSRT was entered as the dependent variable. This model was significant (*F*_(14, 37)_ = 2.33, *p* = 0.02); however, the regression diagnostics were not acceptable, as the linearity assumption was not satisfied. The L_parahippocampal region was then removed because the component-plus-residual-plot showed a non-linear curve with maximum deviation from the linear curve. Following this, the L_precentral, L_IOG, L_SOG and R_angular regions were removed in order. Finally, we obtained the final nine features: L_MFG, R_IFG_oper, L_IFG_tri, R_Rolandic_oper, L_SMA, L_hippocampus, R_lingual, R_postcentral and L_MTG (Table 1 and Figure 5). The model that was then built using these nine features was significant (*F*_(9, 42)_ = 6.80, *p* < 0.0001). The *R*^2^ was 0.994 and the adjusted *R*^2^ was 0.986. The regression diagnostics were acceptable, with all VIFs being under 4. The relative weights of these features were 13.242%, 12.236%, 12.556%, 10.399%, 9.058%, 9.623%, 11.854%, 9.306% and 11.725%, respectively (Figure 6A). In the prediction analysis, the mean r(pred, actual) was 0.948 ± 0.0503 and the mean MSE was 0.0039 ± 0.0011. The MSE was significantly negative correlated with r(pred, actual; *r* = −0.944, *p* < 0.0001, Figure 6B), suggesting that model with the higher r(pred, actual) would show the lower MSE. In order to estimate the overfitting, we calculated the MSE of training set and found that the mean MSE was 0.0011 ± 0.0003. There was little difference between this value and the MSE of test set (0.0039 ± 0.0011). This result suggests that the model built in our study could well fit the training data, but also could be generalized to test data without overfitting.

**Table 1:** Prediction performance using linear regression model.

Threshold	Features	*R*^2^	Adjusted *R*^2^	Weights (%)	MSE	r(pred, actual)
	L_MFG			13.242		
	R_IFG_oper			12.236		
	L_IFG_tri			12.556		
	R_Rolandic_oper			10.399		
*P* < 0.05	L_SMA	0.994	0.986	9.058	0.0039 ± 0.0011	0.948 ± 0.0503
	L_ hippocampus			9.623		
	R_ lingual			11.854		
	R_ postcentral			9.306		
	L_MTG			11.725		
	L_MFG			15.558		
	R_Rolandic_oper			18.738		
*P* < 0.01	L_postcentral	0.966	0.950	25.337	0.0056 ± 0.0017	0.916 ± 0.0236
	R_postcentral			19.650		
	L_supramarginal			20.716		

*R^2^, the coefficient of determination; adjusted R^2^, the adjusted coefficient of determination; MSE, mean square error; r(pred, actual), the Pearson’s correlation coefficient between the predicted values and the actual values; weights, the relative importance of independent variables; L_MFG, left middle frontal gyrus; R_IFG_oper, pars opercularis of right inferior frontal gyrus; L_IFG_tri, pars triangularis of left inferior frontal gyrus; R_Rolandic_oper, pars opercularis of right rolandic area; L_SMA, left supplementary motor area; L_hippocampus, left hippocampus; R_lingual, right lingual gyrus; R_postcentral, right postcentral gyrus; L_MTG, left middle temporal gyrus; L_postcentral, left postcentral gyrus; L_supramarginal, left supramarginal gyrus*.

For *p* < 0.01, we built the linear regression model with 24 regions without LASSO. Using the similar prediction process, five regions were finally selected (Table 1): L_MFG, R_Rolandic_oper, left postcentral gyrus (L_postcentral), R_postcentral and left supramarginal gyrus (L_supramarginal). The model built with these five features was significant (*F*_(5, 46)_ = 10.306, *p* < 0.0001). The *R*^2^ was 0.966 and the adjusted *R*^2^ was 0.950. The relative weights of these features were 15.558%, 18.738%, 25.337%, 19.650%, and 20.716% respectively. The mean r(pred, actual) was 0.916 ± 0.0236 and the mean MSE 0.0056 ± 0.0017. The MSE was significantly negative correlated with r(pred, actual; *r* = −0.961, *p* < 0.0001). However, for *p* < 0.005, the model with 13 regions was not significant (*F*_(13, 38)_ = 1.608, *p* = 0.126). The regression diagnostics of the model for *p* < 0.001 showed that it was not acceptable, with the homoscedasticity assumption not being satisfied. These results suggest that the best prediction performance was at the uncorrected threshold of *p* < 0.05 with higher r(pred, actual) and lower MSE than that using strict regions thresholds.

## Discussion

In the present study, we used a linear regression model to examine the predictive ability of regional GM volumes for determining the difference in the rate of response inhibition before and after SD. First, we investigated the correlation between ΔSSRT and GM volumes of brain regions. We then selected the optimal regions for predicting ΔSSRT using a linear regression model. We found that the GM volumes of the L_MFG, R_IFG_oper, L_IFG_tri, R_Rolandic_oper, L_SMA, L_hippocampus, R_lingual, R_postcentral, and L_MTG could accurately predict ΔSSRT with low MSE and high r(pred, actual). Our results suggest that it is possible to predict the impairment of response inhibition after SD using only structural MRI data.

Behaviorally, we found that SD impaired the performance of response inhibition, and that subjects with faster SSRT after RW showed a greater change in SSRT after SD. These results suggest that the impairment of response inhibition could be qualitatively predicted by the performance after RW. In this study, we investigated the quantitatively prediction performance of structural MRI data in ΔSSRT. First, we investigated the correlation between ΔSSRT and GM volumes of brain regions. No significant correlation was observed between ΔSSRT and GM volume of any brain region when the statistics were corrected for multiple comparisons using FWE correction. However, we found that regional GM volumes could predict the ΔSSRT. This contradictory result may arise from the strict threshold in the voxel based analysis. The former performed the Pearson correlation analysis for each voxel, which increased the false positive. In order to avoid this effect, multiple comparisons using FWE correction was performed which was a strict correction method. In the present study, we selected the nine regions via machine-learning and cross-validation. This procedure includes a built-in guard against false positives (Finn et al., 2015). If the proportion of false positives in the feature-extraction step is high, the regression model is unlikely to generalize well to independent data. Therefore, it is not necessary to correct for multiple comparisons in the VBM analysis when extracting features.

Then, we extracted those regions in which GM volumes were negative correlated with ΔSSRT at uncorrected threshold, to act as input features for a linear regression model. To explore the effect of different thresholds on the prediction, we chose uncorrected thresholds of *p* < 0.001, *p* < 0.005, *p* < 0.01, and *p* < 0.05 (all with a minimum of 10 voxels) to determine the voxels which GM volumes were negative correlated with ΔSSRT. We found that the best prediction performance was obtained at the uncorrected threshold of *p* < 0.05, with higher r(pred, actual) and lower MSE than that with other thresholds. It seems to be taken for granted that the predictive power using regions threshold at *p* < 0.05 outperformed that using less regions threshold at *p* < 0.01 to *p* < 0.001. As more candidate features were involved at *p* < 0.05, and importantly it included all the regions at other strict thresholds, thus the method could of course be more predictive when using regions threshold at *p* < 0.05. However, although the candidate features at *p* < 0.05 included all the regions at other strict thresholds in the feature extraction step, the LASSO was performed to reduce the feature dimensions in the feature selection step. We also manually and gradually excluded the remaining candidate features in the prediction analysis step. The finally selected regions at *p* < 0.05 were not always more than those at other strict thresholds. The number of selected regions was dependent on the data itself. Therefore, the better prediction performance at *p* < 0.05 resulted from the more final selected regions, not the candidate features.

We found that the R_IFG_oper was selected to predict the ΔSSRT. Recent studies have found that the R_IFG_oper was specially engaged in the successful inhibition, and the activation and GM intensity of this region were both negative correlated with SSRT (Congdon et al., 2010; Tabibnia et al., 2011; Aron et al., 2014), consistent with the critical role of R_IFG_oper in inhibition control. Therefore, it is reasonable that this region was involved in the prediction model. However, we didn’t select the right MFG, SMA or MTG as the final features. This finding does not conflict with reports of neural activation during inhibitory control, as greater GM intensity in a region is not necessarily coupled with greater or lower activation in that region. Although the right-hemisphere dominance in activation is well-known, some studies have also reported bilateral activation in IFG, MFG, SMA and MTG (Li et al., 2006; Cohen et al., 2010; Congdon et al., 2014). Furthermore, Lee and Hsieh (2017) have found that the regional homogeneity/fractional amplitude of low-frequency fluctuation and SSRT were significantly negative correlated in the left IFG. The L_MFG and L_IFG_tri contributed the most to the coefficient of determination of the linear regression model in our present study. These findings suggest that left IFG and left MFG may also play an important role in response inhibition. We also selected the R_lingual as a predictor of ΔSSRT. During the SST part of the study, participants were required to cancel a button press when the white arrow changed to blue after a visual stimulus. This part of the task means that the lingual gyrus is likely to be involved in response inhibition, as this region plays a role in visual memories (Jeneson et al., 2012) and color vision processing (Bogousslavsky et al., 1987). Although previous studies do not find the engagement of lingual gyrus, the occipital cortex was implicated. These results suggest that the vision-related region may predict the ΔSSRT. These findings indicate that these regions consisting of the “stopping network” could predict the ΔSSRT, consistent with our hypothesis. An interesting finding is that the R_Rolandic_oper, L_hippocampus and R_postcentral were also involved in the prediction model. This result suggests that the combination of the stopping network, somatosensory cortex and hippocampus could predict the ΔSSRT accurately.

In our study, a traditional linear regression model was built to predict the ΔSSRT. However, the dimension of the independent variables should be less than the sample size in such a linear regression model. LASSO was therefore performed to reduce the feature dimensions and select the independent variables with the strongest explanatory power for the dependent variable. To investigate the effect of the dimensional reduction on prediction performance, we used support vector regression (SVR), a machine-learning based multiple regression method (Drucker et al., 1996; Smola and Schölkopf, 2004), to predict the ΔSSRT. This method found the hyperplane with a maximum margin to minimize the error, keeping in mind that part of the error is tolerated and broke the limitation of the linear regression model (i.e., the dimension of the independent variables in SVR could be more than the sample size). A range of uncorrected thresholds were also chosen (*p* < 0.05, 0.01, 0.005 and 0.001, with a minimum of 10 voxels) to determine the voxels which GM volumes were negative correlated with ΔSSRT. We found that the best prediction performance was obtained at an uncorrected threshold of *p* < 0.01, with an r(pred, actual) = 0.995 (*p* < 0.0001) and an MSE = 0.0065 being obtained (Supplementary Table S5). Compared with the linear regression model, SVR tended to produce higher r(pred, actual), but also larger MSE. It is hard to determine which model is better, however, we found that the increase rate of MSE was larger than that of r(pred, actual) between these two models. These results suggest that the linear regression model may be more appropriate for predicting ΔSSRT.

There are several limitations to the present study that require consideration. First, the sample size in this study was relatively small; thus, the findings should be replicated in a larger sample. Second, we used a single modality to extract features. Multimodality imaging (VBM, DTI and resting-state fMRI) may result in better prediction performance. Further studies are expected to test these hypotheses. Third, we only recruited the young subjects, except the older adults in this present study. However, Hu et al. (2014) found that aging is significantly positive correlated with SSRT among 18–72 years adults and is associated with decreased GM volume. Therefore, the linear regression model in young subjects may be not identical with that in older subjects. Aging may affect the prediction performance of GM volume. Considering this factor, we only recruited the young subjects in our present study. On the other hand, we also attempt to explore the structural basis of SD effect on SSRT. In order to exclude the age factor, we only analyzed the young subjects. Further studies with larger sample size and more comprehensive sample are required to examine the predictive ability of regional GM volumes.

In conclusion, our results demonstrate that structural MRI data can be used for the quantitative prediction of the change in response inhibition performance after SD.

## Author Contributions

JS, PL, XY and WQ contributed to the conception and design. RZ, XZ, YZ and NF contributed to the acquisition of data, data analysis and manuscript writing. JS, PL, XY and RZ contributed to the interpretation of the results.

## Conflict of Interest Statement

The authors declare that the research was conducted in the absence of any commercial or financial relationships that could be construed as a potential conflict of interest.

## Footnotes

^1^http://www.fmrib.ox.ac.uk/fsl/

^2^www.fmrib.ox.ac.uk/analysis/techrep

^3^http://www.r-project.org/
