# Supplementary material for: Prediction of the Effect of Sleep Deprivation on Response Inhibition via Machine Learning on Structural Magnetic Resonance Imaging Data
Source: Front Hum Neurosci. 2018 Jul 10;12:276. doi: 10.3389/fnhum.2018.00276 (PMC6048191; doi:10.3389/fnhum.2018.00276)
Supplement: Supplementary file 1 [file Table_1.DOCX]

**Supplementary Table 1. Significant correlation between grey matter volume and △SSRT at uncorrected threshold p<0.05 with the minimum cluster size of 10 voxels**

| **Regions** | **Hemisphere** | **Min p** | **X** | **Y** | **Z** | **Voxels** |
| --- | --- | --- | --- | --- | --- | --- |
| **Precentral gyrus** | L | 0.0040 | -30 | 4 | 50 | 683 |
|  | R | 0.0028 | 52 | -2 | 22 | 533 |
| **Superior frontal gyrus** | L | 0.0040 | -28 | -2 | 62 | 163 |
|  | R | 0.0080 | 14 | -8 | 60 | 57 |
| **Superior frontal gyrus, orbital part** | R | 0.0334 | 10 | 18 | -24 | 15 |
| **Middle frontal gyrus** | L | 0.0044 | -34 | 12 | 40 | 455 |
| **Inferior frontal gyrus, opercular part** | L | 0.0118 | -46 | 12 | 16 | 84 |
|  | R | 0.0032 | 44 | 18 | 8 | 169 |
| **Inferior frontal gyrus, triangular part** | L | 0.0056 | -38 | 36 | -2 | 100 |
|  | R | 0.0106 | 44 | 26 | 12 | 146 |
| **Inferior frontal gyrus, orbital part** | L | 0.0150 | -38 | 36 | -4 | 36 |
| **Rolandic operculum** | L | 0.0006 | -54 | -18 | 22 | 198 |
|  | R | 0.0030 | 50 | -8 | 18 | 397 |
| **Supplementary motor area** | L | 0.0106 | -8 | -10 | 58 | 254 |
|  | R | 0.0130 | 12 | -16 | 52 | 259 |
| **Olfactory cortex** | R | 0.0356 | 4 | 10 | -16 | 11 |
| **Gyrus rectus** | R | 0.0294 | 8 | 18 | -26 | 23 |
| **Insula** | L | 0.0178 | -44 | -2 | 6 | 83 |
|  | R | 0.0242 | 32 | -22 | 18 | 119 |
| **Middle cingulate cortex** | L | 0.0124 | -14 | -22 | 42 | 113 |
|  | R | 0.0342 | 2 | -20 | 44 | 30 |
| **Posterior cingulate cortex** | L | 0.0274 | 0 | -54 | 30 | 18 |
|  | R | 0.0216 | 4 | -46 | 10 | 54 |
| **Hippocampus** | L | 0.0272 | -30 | -12 | -22 | 40 |
| **ParaHippocampal gyrus** | L | 0.0234 | -12 | -4 | -30 | 105 |
|  | R | 0.0320 | 14 | -8 | -24 | 39 |
| **Amygdala** | R | 0.0306 | 16 | 0 | -16 | 13 |
| **Calcarine sulcus** | L | 0.0204 | -14 | -90 | 0 | 218 |
|  | R | 0.0044 | 20 | -88 | -2 | 121 |
| **Cuneus** | L | 0.0034 | -14 | -72 | 36 | 148 |
|  | R | 0.0052 | 6 | -88 | 38 | 398 |
| **Lingual gyrus** | L | 0.0282 | -6 | -96 | -18 | 44 |
|  | R | 0.0026 | 20 | -80 | -4 | 156 |
| **Superior occipital cortex** | L | 0.0020 | -18 | -70 | 36 | 97 |
|  | R | 0.0012 | 26 | -74 | 30 | 812 |
| **Middle occipital cortex** | L | 0.0172 | -26 | -66 | 30 | 248 |
|  | R | 0.0018 | 30 | -74 | 32 | 638 |
| **Inferior occipital cortex** | L | 0.0188 | -40 | -64 | -4 | 46 |
| **Fusiform gyrus** | L | 0.0146 | -38 | -52 | -14 | 213 |
|  | R | 0.0368 | 48 | -56 | -14 | 16 |
| **Postcentral gyrus** | L | 0.0002 | -56 | -22 | 52 | 1811 |
|  | R | 0.0022 | 54 | -4 | 22 | 376 |
| **Superior parietal gyrus** | L | 0.0004 | -28 | -66 | 58 | 560 |
|  | R | 0.0016 | 26 | -80 | 52 | 318 |
| **Inferior parietal lobule** | L | 0.0004 | -46 | -38 | 40 | 718 |
|  | R | 0.0066 | 34 | -34 | 50 | 128 |
| **SupraMarginal gyrus** | L | 0.0006 | -54 | -22 | 20 | 377 |
|  | R | 0.0090 | 48 | -46 | 34 | 111 |
| **Angular gyrus** | L | 0.0092 | -46 | -50 | 32 | 34 |
|  | R | 0.0004 | 46 | -46 | 32 | 690 |
| **Precuneus** | L | 0.0016 | -6 | -64 | 30 | 913 |
|  | R | 0.0088 | 18 | -74 | 40 | 294 |
| **Paracentral lobule** | L | 0.0024 | -8 | -32 | 74 | 145 |
| **Heschl gyrus** | L | 0.0052 | -56 | -2 | 6 | 16 |
|  | R | 0.0182 | 52 | -16 | 12 | 29 |
| **Superior temporal gyrus** | L | 0.0004 | -58 | -22 | 16 | 392 |
|  | R | 0.0076 | 68 | -10 | 12 | 186 |
| **Middle temporal gyrus** | L | 0.0270 | -40 | -60 | -2 | 37 |
|  | R | 0.0062 | 46 | -52 | 16 | 137 |
| **Inferior temporal gyrus** | L | 0.0226 | -60 | -48 | -24 | 181 |
|  | R | 0.0084 | 50 | -52 | -8 | 107 |
