# Supplementary material for: Prediction of the Effect of Sleep Deprivation on Response Inhibition via Machine Learning on Structural Magnetic Resonance Imaging Data
Source: Front Hum Neurosci. 2018 Jul 10;12:276. doi: 10.3389/fnhum.2018.00276 (PMC6048191; doi:10.3389/fnhum.2018.00276)
Supplement: Supplementary file 2 [file Table_2.DOCX]

**Supplementary Table 2. Significant correlation between grey matter volume and △SSRT at the uncorrected threshold p<0.01 with the minimum cluster size of 10 voxels**

| **Regions** | **Hemisphere** | **Min p** | **X** | **Y** | **Z** | **Voxels** |
| --- | --- | --- | --- | --- | --- | --- |
| **Precentral gyrus** | L | 0.0040 | -30 | 4 | 50 | 33 |
|  | R | 0.0028 | 52 | -2 | 22 | 41 |
| **Middle frontal gyrus** | L | 0.0044 | -34 | 12 | 40 | 37 |
| **Inferior frontal gyrus, opercular part** | R | 0.0032 | 44 | 18 | 8 | 21 |
| **Inferior frontal gyrus, triangular part** | L | 0.0056 | -38 | 36 | -2 | 11 |
| **Rolandic operculum** | L | 0.0006 | -54 | -18 | 22 | 53 |
|  | R | 0.0030 | 50 | -8 | 18 | 38 |
| **Cuneus** | L | 0.0034 | -14 | -72 | 36 | 47 |
|  | R | 0.0052 | 6 | -88 | 38 | 46 |
| **Lingual gyrus** | R | 0.0026 | 20 | -80 | -4 | 26 |
| **Superior occipital cortex** | L | 0.0020 | -18 | -70 | 36 | 25 |
|  | R | 0.0012 | 26 | -74 | 30 | 396 |
| **Middle occipital cortex** | R | 0.0018 | 30 | -74 | 32 | 127 |
| **Postcentral gyrus** | L | 0.0002 | -56 | -22 | 52 | 894 |
|  | R | 0.0022 | 54 | -4 | 22 | 18 |
| **Superior parietal gyrus** | L | 0.0004 | -28 | -66 | 58 | 183 |
|  | R | 0.0016 | 26 | -80 | 52 | 55 |
| **Inferior parietal lobule** | L | 0.0004 | -46 | -38 | 40 | 416 |
| **SupraMarginal gyrus** | L | 0.0006 | -54 | -22 | 20 | 169 |
| **Angular gyrus** | R | 0.0004 | 46 | -46 | 32 | 80 |
| **Precuneus** | L | 0.0016 | -6 | -64 | 30 | 319 |
| **Paracentral lobule** | L | 0.0024 | -8 | -32 | 74 | 40 |
| **Superior temporal gyrus** | L | 0.0004 | -58 | -22 | 16 | 156 |
| **Middle temporal gyrus** | R | 0.0062 | 46 | -52 | 16 | 14 |
