# Supplementary material for: Prediction of the Effect of Sleep Deprivation on Response Inhibition via Machine Learning on Structural Magnetic Resonance Imaging Data
Source: Front Hum Neurosci. 2018 Jul 10;12:276. doi: 10.3389/fnhum.2018.00276 (PMC6048191; doi:10.3389/fnhum.2018.00276)
Supplement: Supplementary file 3 [file Table_3.DOCX]

**Supplementary Table 3. Significant correlation between grey matter volume and △SSRT at uncorrected threshold p<0.005 with the minimum cluster size of 10 voxels**

| **Regions** | **Hemisphere** | **Min p** | **X** | **Y** | **Z** | **Voxels** |
| --- | --- | --- | --- | --- | --- | --- |
| **Rolandic operculum** | L | 0.0006 | -54 | -18 | 22 | 28 |
| **Superior occipital cortex** | L | 0.0020 | -18 | -70 | 36 | 16 |
|  | R | 0.0012 | 26 | -74 | 30 | 209 |
| **Middle occipital cortex** | R | 0.0018 | 30 | -74 | 32 | 49 |
| **Postcentral gyrus** | L | 0.0002 | -56 | -22 | 52 | 407 |
| **Superior parietal gyrus** | L | 0.0004 | -28 | -66 | 58 | 79 |
|  | R | 0.0016 | 26 | -80 | 52 | 21 |
| **Inferior parietal lobule** | L | 0.0004 | -46 | -38 | 40 | 280 |
| **SupraMarginal gyrus** | L | 0.0006 | -54 | -22 | 20 | 89 |
| **Angular gyrus** | R | 0.0004 | 46 | -46 | 32 | 19 |
| **Precuneus** | L | 0.0016 | -6 | -64 | 30 | 150 |
| **Paracentral lobule** | L | 0.0024 | -8 | -32 | 74 | 20 |
| **Superior temporal gyrus** | L | 0.0004 | -58 | -22 | 16 | 81 |
