# Supplementary material for: Prediction of the Effect of Sleep Deprivation on Response Inhibition via Machine Learning on Structural Magnetic Resonance Imaging Data
Source: Front Hum Neurosci. 2018 Jul 10;12:276. doi: 10.3389/fnhum.2018.00276 (PMC6048191; doi:10.3389/fnhum.2018.00276)
Supplement: Supplementary file 4 [file Table_4.DOCX]

**Supplementary Table 4. Significant correlation between grey matter volume and △SSRT at uncorrected threshold p<0.001 with the minimum cluster size of 10 voxels**

| **Regions** | **Hemisphere** | **Min p** | **X** | **Y** | **Z** | **Voxels** |
| --- | --- | --- | --- | --- | --- | --- |
| **Postcentral gyrus** | L | 0.0002 | -56 | -22 | 52 | 68 |
| **Superior parietal gyrus** | L | 0.0004 | -28 | -66 | 58 | 13 |
| **Inferior parietal lobule** | L | 0.0004 | -46 | -38 | 40 | 36 |
| **SupraMarginal gyrus** | L | 0.0006 | -54 | -22 | 20 | 12 |
