# Supplementary material for: Prediction of the Effect of Sleep Deprivation on Response Inhibition via Machine Learning on Structural Magnetic Resonance Imaging Data
Source: Front Hum Neurosci. 2018 Jul 10;12:276. doi: 10.3389/fnhum.2018.00276 (PMC6048191; doi:10.3389/fnhum.2018.00276)
Supplement: Supplementary file 5 [file Table_5.DOCX]

**Supplementary Table 5.** **Prediction performance using SVR model.**

| **Threshold** | **Number of Features** | **MSE** | **r(pred,actual)** | **p** |
| --- | --- | --- | --- | --- |
| **P<0.05** | 61 | 0.0108 | 0.996 | <0.0001 |
| **P<0.01** | 24 | 0.0065 | 0.995 | <0.0001 |
| **P<0.005** | 13 | 0.0203 | 0.963 | <0.0001 |
| **P<0.001** | 4 | 0.0096 | 0.873 | <0.0001 |

MSE, mean square error, the average of the squares of the difference between the predicted value and the actual value; r(pred,actual), the Pearson’s correlation coefficient between the predicted values and the actual values; p, the significant of the Pearson’s correlation.
